# Supplementary material for: Phenotypic and Genotypic Characterization of ESBL-, AmpC-, and Carbapenemase-Producing Klebsiella pneumoniae and High-Risk Escherichia coli CC131, with the First Report of ST1193 as a Causative Agent of Urinary Tract Infections in Human Patients in Algeria
Source: Antibiotics (Basel). 2025 May 9;14(5):485. doi: 10.3390/antibiotics14050485 (PMC12108494; doi:10.3390/antibiotics14050485)
Supplement: Supplementary file 1 [file antibiotics-14-00485-s001.zip › antibiotics-3575826_SupplementaryTables_proofread.pdf]

antibiotics

MDPI

Supplementary Materials

Phenotypic and Genotypic Characterization of ESBL-, AmpC-, and Carbapenemase-Producing *Klebsiella pneumoniae* and High-Risk *Escherichia coli* ST131, with the First Report of ST1193 as a Causative Agent of Urinary Tract Infections in Human Patients in Algeria

Hajer Ziadi, Fadela Chougrani, Abderrahim Cheriguene, Leticia Carballeira, Vanesa García, Azucena Mora

Correspondence: azucena.mora@usc.es; vanesag.menendez@usc.es

Table S3. Pairwise distance matrix calculated from SNP in the Algerian and Spanish ST1193 genomes

Column1	LREC_265	LREC_270	LREC_273	LREC_275	LREC_468	LREC_269 (Reference)
LREC_265	0	114	90	75	82	72
LREC_270	114	0	136	121	128	118
LREC_273	90	136	0	97	96	94
LREC_275	75	121	97	0	83	73
LREC_468	82	128	96	83	0	78
LREC_269 (Reference)	72	118	94	73	78	0

min: 72 max: 136

Table S4. Pairwise distance matrix calculated from SNP in the Algerian, Spanish and ESC_RA5887AA ST1193 genomes

	ESC_RA5887AA	LREC_265	LREC_270	LREC_273	LREC_275	LREC_468	LREC_269 (Reference)
ESC_RA5887AA	0	68	182	83	75	55	65
LREC_265	68	0	188	95	95	75	73
LREC_270	182	188	0	205	201	191	189
LREC_273	83	95	205	0	102	102	98
LREC_275	75	95	201	102	0	88	84
LREC_468	55	75	191	102	88	0	60
LREC_269 (Reference)	65	73	189	98	84	60	0

min: 55 max: 205

Table S5. Targets and primers used for *Escherichia coli* and *Klebsiella pneumoniae* identification

**Target**	**Primers**	**Nucleotide sequence (5´- 3´)**	**Size (bp)**	**Annealing T (°C)**	**Reference**
uidA	uidA-F	GCGTCTGTTGACTGGCAGGTGGTGG	503	60	[22]
	uidA-R	GTTGCCCGCTTCGAAACCAATGCCT			
kp50233	50233-F	GCTCTGGGAGATAGACCGCA	484	67	[82]
	50233-R	GCGATSGCAGACCAGATGAAT			

Table S6. Primers used for the detection and/or Sanger sequencing of *bla* genes

Target	Primers	Nucleotide sequence (5´- 3´)	Size (bp)	Annealing T (°C)	Reference
*bla*CTX-M	CTX-C3	ATGTGCAGCACCAGTAAAGTGATG	542	55	[87]
	CTX-C4	ACCGCGATATCGTTGGTGGTGCC			
*bla*CTX-M group1	M13U	GGTTAAAAAATCACTGCGTC	863	60	[88]
	M13L	TTGGTGACGATTTTAGCCGC			
*bla*CTX-M group1	[a]CTX-15-F1	GAAGCTAATAAAAAACACACGTGG	1044-1123	52	[87]
	[a]CTX-15-R	GTATGCGCAAGCGCAGGTGG			
blaCTX-M group9	CTX-M9-F	GTGACAAAGAGAGTGCAACGG	856	64	[89]
	CTX-M9-R	ATGATTCTCGCCGCTGAAGCC			
blaCTX-M group9	[a]CTX-M9-14-14B-24F	GAATACTGATGTAACACGGA	998	44	[90]
	[a]CTX-M9-R	AGCTGAAGATGTATATCAAG			
*bla*CTX-M group9	[a]CTX-M9-14-14B-24F	GAATACTGATGTAACACGGA	989	52	[90]
	[a]CTX-M14-24-R	CTGCGTTGTCGGGAAGATACG			
*bla*CTX-M group9	[a]CTX-M9-14B-F	CCTATACCCGAGGCGCGACAG	1059	44	[90]
	[a]CTX-M9-R	AGCTGAAGATGTATATCAAG			
*bla*CTX-M group9	[a]CTX-M14-24-F	CTAAATTCTTCGTGAAATAGTG	1049	44	[90]
	[a]CTX-M14-24-R	CTGCGTTGTCGGGAAGATACG			
*bla*SHV	[b]SHV-F2	TTGTCGCTTCTTTACTCGCC	879	64	[87]
	[b]SHV-R2	CCCGGCGATTTGCTGATTTCGC			
LAT-1 to LAT-4, CMY-2 to CMY-7, BIL-1	[b]CITMF	TGGCCAGAACTGACAGGCAAA	462	66	[91]
	[b]CITMR	TTTCTCCTGAACGTGGCTGGC			
*bla*KPC	KPC-Fm	CGTCTAGTTCTGCTGTCTTG	798	56	[86]
	KPC-Rm	CTTGTCATCCTTGTTAGGCG			
*bla*NDM	NDM-F	GGTTTGGCGATCTGGTTTTC	621	58	[86]
	NDM-R	CGGAATGGCTCATCACGATC			
*bla*OXA-48	OXA-F	GCGTGGTTAAGGATGAACAC	438	56	[86]
	OXA-R	CATCAAGTTCAACCCAACCG			
*bla*IMP	VIM-F	GATGGTGTTTGGTCGCATA	390	58	[86]
	VIM-R	CGAATGCGCAGCACCAG			
blaVIM	IMP-F	GGAATAGAGTGGCTTAAYTCTC	232		
	IMP-R	GGTTTAAYAAAACAACCACC			

[a]Primers used for sequencing. [b]Primers used for screening and sequencing.

Table S7. Primers used for the detection of *mcr* genes

Target	Primers	Nucleotide sequence (5´- 3´)	Size (bp)	Annealing T (°C)	Reference
mcr-1	mcr1_320bp_fw	AGTCCGTTTGTTCTTGTGGC	320	58	[92]
	mcr1_320bp_rev	AGATCCTTGGTCTCGGCTTG			
mcr-2	mcr2_700bp_fw	CAAGTGTGTTGGTCGCAGTT	715		[92]
	mcr2_700bp_rev	TCTAGCCCGACAAGCATACC			
mcr-3	mcr3_900bp_fw	AAATAAAAATTGTTCCGCTTATG	929		[92]
	mcr3_900bp_rev	AATGGAGATCCCCGTTTTT			
mcr-4	mcr4_1100bp_fw	TCACTTTCATCACTGCGTTG	1116		[92]
	mcr4_1100bp_rev	TTGGTCCATGACTACCAATG			
mcr-5	MCR5_FW	ATGCGGTTGTCTGCATTTATC	1644		[93]
	MCR5_RV	TCATTGTGGTTGTCCTTTTCTG			

Table S8. Targets and primers used to determine the UPEC and ExPEC status

Target	Primers	Nucleotide sequence (5´- 3´)	Size (bp)	Annealing T (°C)	Reference
UPEC Status					
vat	vat-F	TCAGGACACGTTCAGGCATTCAGT	1100	70	[24]
	vat-R	GGCCAGAACATTTGCTCCCTTGTT			
chuA	chuA-F	CTGAAACCATGACCGTTACG	652	63	
	chuA-R	TTGTAGTAACGCACTAAACC			
fyuA	fyuA-F	GTAAACAATCTTCCCGCTCGGCAT	850	63	
	fyuA-R	TGACGATTAACGAACCGGAAGGGA			
yfcV	yfcV-F	ACATGGAGACCACGTTCACC	292	63	
	yfcV-R	GTAATCTGGAATGTGGTCAGG			
ExPEC status					
papAH	papAH-f	ATGGCAGTGGTGTCTTTTGGTG	720	68	[33]
	papAH-r	CGTCCCACCATACGTGCTCTTC			
sfa/foc	sfa 1	CTCCGGAGAACTGGGTGCATCTTAC	410	64	[94]
	sfa 2	CGGAGGAGTAATTACAAACCTGGCA			
afa/draBC	afa1	GCTGGGCAGCAAACTGATAACTCTC	750	54	
	afa2	CATCAAGCTGTTTGTTCGTCCGCCG			
iutA	aer-851F	GGCTGGACATCATGGGAACTGG	301	60	[33]
	aer-1152R	CGTCGGGAACGGGTAGAATCG			
kpsMII	KpsII f	GCGCATTTGCTGATACTGTTG	272	60	[33]
	KpsII r	CATCCAGACGATAAGCATGAGCA			

Table S9. Targets and primers used for the phylogroup determination in *E. coli*

Target	Primers	Nucleotide sequence (5′- 3′)	Size (bp)	Annealing Tª (°C)	Reference
chuA	chuA.1b	ATGGTACCGGACGAACCAAC	288	58	[26,27]
	chuA.2	TGCCGCCAGTACCAAAGACA			
yjaA	yjaA.1b	CAAACGTGAAGTGTCAGGAG	211		[26]
	yjaA.2b	AATGCGTTCCTCAACCTGTG			
TspE4C2	TspE4C2.1b	CACTATTCGTAAGGTCATCC	152		[26]
	TspE4C2.2b	AGTTTATCGCTGCGGGTCGC			
arpA	AceK.f	AACGCTATTCGCCAGCTTGC	400		[26]
	ArpA1.r	TCTCCCCATACCGTACGCTA			
trpAgpC (C)	trpAgpC.1	AGTTTTATGCCCAGTGCGAG	219	56	[95]
	trpAgpC.2	TCTGCGCCGGTCACGCCC			
arpA (E)	ArpAgpE.f	GATTCCATCTTGTCAAAATATGCC	301	57	[95]
	ArpAgpE.r	GAAAAGAAAAAGAATTCCCAAGAG			
trpA	trpBA.f	CGGCGATAAAGACATCTTCAC	489	56	[28,29]
	trpBA.r	GCAACGCGGCCTGGCGGAAG			
ybgD (G)	ybgD.1	TATGCGGCTGATGAAGGATC	177	59	[27]
	ybgD.2	GTTGACTAAGCGCAGGTCGA			
cfaB (F)	cfaB.1	CTAACGTTGATGCTGCTCTG	384		[27]
	cfaB.2	TGCTAACTACGCCACGGTAG			

Table S10. Targets and primers to determine clonotypes (CH) by Sanger sequencing

Target	Primers	Nucleotide sequence (5′- 3′)	Size (bp)	Annealing T (°C)	Reference
fumC	fumC-F	TCACAGGTCGCCAGCGCTTC	469	56	[98]
	fumC-R	GTACGCAGCGAAAAAGATTC			
fimH	fimH-F	CACTCAGGGAACCATTCAGGCA	489	54	[31]
	fimH-R	CTTATTGATAAACAAAAGTCAC			

Allele assignments for *fumC* and *fimH* were determined using the CHTyper database available at the Center for Genomic Epidemiology (CGE) website https://cge.food.dtu.dk/services/CHTyper/.

Table S11. Primers used for the *rbf*O25, H4 (*fliC*H4) and H5 (*fliC*H5) screening

Target	Primers	Nucleotide sequence (5´- 3´)	Size (bp)	Annealing T (°C)	Reference
*rfb*O25b	rfbO25b.r	TGCTATTCATTATGCGCAGC	300	56	[26,27]
	rfb.1bis	ATACCGACGACGCCGATCTG			
*fliC*H4	H4-F	GCAGCGTATTCGTGAACTGA	713	66	[96]
	H4-R	GCTGGATAATCTGCGCTTTC			
*fliC*H5	H5-F2	GGATGAAATTGATCGCGTTT	477	48-52	[10]
	H5-R	GTAGCCGCAGTCGTTAGTCC			

Table S12. Targets and primers used in the virotype scheme of CC131 isolates

Target	Primers	Nucleotide sequence (5´- 3´)	Size (bp)	Annealing Tª (°C)	Reference
afa/draBC	afa1	GCTGGGCAGCAAACTGATAACTCTC	750	64	[94]
	afa2	CATCAAGCTGTTTGTTCGTCCGCCG			
afaFM955459	Afa-025F	GAGTCACGGCAGTCGCGGCGG	207	55	[28]
	Afa-025R	TTCACCGGCGACCAGCCATCTCC			
iroN	Ironec-f	AAGTCAAAGCAGGGGTTGCCCG	665	62	[97]
	Ironec-r	GACGCCGACATTAAGACGCAG			
sat	SatF	GCAGCTACCGCAATAGGAGGT	937	60	[98]
	SatR	CATTCAGAGTACCGGGGCCTA			
ibeA	Ibe10 f	AGGCAGGTGTGCGCCGCGTAC	170	58	[33]
	Ibe10 r	TGGTGCTCCGGCAAACCATGC			
papGII	Pap-II f	GGGCATTGCTACGGTAACCTG	545	54-60	[87]
	Pap-II r	CGCTATTAATAGACAGATCACC			
papGIII	Pap-III f	CGGCAACTTTAAGCTATGTG	720	60-68	[87]
	Pap-III r	TGTACCATCTCATCGTTGTCTC			
cnf1	CNF1-F2	CAGGAGGTACTTAGCAGCGT	468	48-58	[87]
	CNF1-RC	TAATTTTGGGTTTGTATC			
hlyA	hly f	AACAAGGATAAGCACTGTTCTGGCT	1177	64	[99]
	hly r	ACCATATAAGCGGTCATTCCCGTCA			
cdtB	cdt-s1	GAAAGTAAATGGAATATAAATGTCCG	466	48-52	[100]
	cdt-as1	AAATCTCCTGCAATCATCCAGTTA			
	cdt-s2	GAAAATAAATGGAACACACATGTCCG			
	cdt-as2	AAATCACCAAGAATCATCCAGTTA			
neuC-K1	neu1	AGGTGAAAAGCCTGGTAGTGTG	676	54	[101]
	neu2	GGTGGTACATCCCGGGATGTC			
kps-M II-K2	kpsII f	GCGCATTTGCTGATACTGTTG	570	60	[102]
	KpsII-K2r	AGGTAGTTCAGACTCACACCT			
kps-M II-K5	K5 f	CAGTATCAGCAATCGTTCTGTA	159	54	[33]
	kpsII r	CATCCAGACGATAAGCATGAGCA			

antibiotics

MDPI

Table S13. Virotype designation scheme for CC131 *E. coli* (adapted from Dahbi et al., 2014) [30].

VIROTYPES	*afa/draBC*	*afa operon FM955459*	*iroN*	*sat*	*ibeA*	*papG II*	*papG III*	*cnf1*	*hlyA*	*cdtB*	*neuCK1*	*kpsM II-K2*	*kpsM II-K5*
Virotype A	+	+	-	+/-	-	-	-	-	-	-	-	+	-
Virotype B	-	-	+	+/-	-	+/-	-	-	-	-	-	-	+/-
Virotype C1	-	-	-	+	-	-	-	-	-	-	-	+	-
Virotype C2	-	-	-	+	-	-	-	-	-	-	-	-	+
Virotype C3	-	-	-	+	-	-	-	-	-	-	-	-	-
Virotype D1	-	-	+/-	-	+	-	-	-	-	+	-	-	+
Virotype D2	-	-	+/-	-	+	-	+	-	-	+	-	-	+
Virotype D3	+/-	+/-	+/-	+/-	+	-	-	-	-	-	-	-	+
Virotype D4	-	-	+/-	-	+	-	-	-	-	-	+	-	-
Virotype D5	-	-	+/-	-	+	-	+	+	+	-	-	-	+
Virotype E	-	-	-	+	-	+	-	+	+	-	-	-	+
Virotype F	-	-	-	+	-	+	-	-	-	-	-	-	+

Table S14. Targets and primers used for virulence genes of *Klebsiella pneumoniae*

Target	Primers	Nucleotide sequence (5´- 3´)	Size (bp)	Annealing Tª (°C)	Reference
iucA	iucA-F2	GCTTATTTCTCCCCAACCC	583	59	[36]
	iucA-R2	TCAGCCCTTTAGCGACAAG			
iroB	iroB-F2	CAAAAAAGCAGCAGAGGC	585	59	
	iroB-R2	TCACTGGCGGAATCCAACAC			
terB	terB-F1	TATCGCTGTTGCCAGTGAC	288	59	
	terB-R1	CGGACAGCACTCTTCTCATC			
peg-344	peg-344-F2	AAAGGACAGAAAGCCAGTG	411	53	
	peg-344-R2	CAATGACGAGGGGGATAATC			
peg-589	peg-589-F1	TGAACCCCTGAAGGTCTATC	236	55	
	peg-589-R1	GTGATGAATAAACTACTGCGGC			
peg-1631	peg-1631-F1	GGGATTTATCAACCGCTTTG	503	59	
	peg-1631-R1	TCTCCAGCATCATCGTCA			
rmpA	prmpA-F	GAGTAGTTAATAAATCAATAGCAAT	332	50	
	prmpA-R	CAGTAGGCATTGCAGCA			
rmpA2	prmpA2-F	GTGCAATAAGGATGTTACATTA	430	50	
	prmpA2-R	GGATGCCCTCCTCCTG			

References

10. García-Meniño, I.; García, V.; Lumbreras-Iglesias, P.; Fernández, J.; Mora, A. Fluoroquinolone resistance in complicated urinary tract infections: association with the increased occurrence and diversity of *Escherichia coli* of clonal complex 131, together with ST1193. *Front Cell Infect Microbiol*. **2024**; 14, 1351618. https://doi.org/10.3389/fcimb.2024.1351618.
22. Gómez-Duarte, O.G.; Arzuza, O.; Urbina, D.; Bai, J.; Guerra, J.; Montes, O.; Puello, M.; Mendoza, K.; Castro, G.Y. Detection of *Escherichia coli* enteropathogens by multiplex polymerase chain reaction from children's diarrheal stools in two Caribbean-Colombian cities. Foodborne *Pathog Dis*. **2010**; 7(2):199-206. https://doi.org/10.1089/fpd.2009.0355.
24. Spurbeck, R. R.; Dinh, P. C.; Walk, S. T.; Stapleton, A. E.; Hooton, T. M.; Nolan, L. K.; Kim, K. S.; Johnson, J. R.; Mobley, H. L. T. *Escherichia coli* isolates that carry *vat, fyuA, chuA,* and *yfcV* efficiently colonize the urinary tract. *Infect Immun*. **2012**; 80(12), 4115–4122. https://doi.org/ 10.1128/IAI.00752-12.
26. Clermont, O.; Christenson, J.K.; Denamur, E.; Gordon, D.M. The Clermont *Escherichia coli* phylo-typing method revisited: improvement of specificity and detection of new phylo-groups. *Environ Microbiol Rep*. **2013**;5, 58-65. https://doi.org/ 10.1111/1758-2229.12019.
27. Clermont, O.; Dixit, O.V.A.; Vangchhia, B.; Condamine, B.; Dion, S.; Bridier-Nahmias, A.; Denamur, E.; Gordon, D. Characterization and rapid identification of phylogroup G in *Escherichia coli*, a lineage

with high virulence and antibiotic resistance potential. *Environ Microbiol*. **2019**, 21(8):3107-3117. https://doi.org/ 10.1111/1462-2920.14713.

28. Blanco, M.; Alonso, M. P.; Nicolas-Chanoine, M.-H.; Dahbi, G.; Mora, A.; Blanco, J. E.; López, C.; Cortés, P.; Llagostera, M.; Leflon-Guibout, V.; et al. Molecular epidemiology of *Escherichia coli* producing extended-spectrum β-lactamases in Lugo (Spain): dissemination of clone O25b:H4-ST131 producing CTX-M-15. *J Antimicrob Chemother*. **2009**; 63(6), 1135–1141. https://doi.org/10.1093/jac/dkp122.
30. Dahbi, G.; Mora, A.; Mamani, R.; López, C.; Alonso, M. P.; Marzoa, J.; Blanco, M.; Herrera, A.; Viso, S.; García-Garrote, F.; et al. Molecular epidemiology and virulence of *Escherichia coli* O16:H5-ST131: comparison with H30 and H30-Rx subclones of O25b:H4-ST131. *Int J Med Microbiol*. **2017**; 304(8), 1247–1257. https://doi.org/10.1016/j.ijmm.2014.10.002
31. Weissman, S. J.; Johnson, J. R.; Tchesnokova, V.; Billig, M.; Dykhuizen, D.; Riddell, K.; Rogers, P.; Qin, X.; Butler-Wu, S.; Cookson, B. T.; et al. High-resolution two-locus clonal typing of extraintestinal pathogenic Escherichia coli. *Appl Environ Microbiol*. **2012**; 78(5):1353-60. https://doi.org/ 10.1128/AEM.06663-11.
33. Johnson, J. R.; Stell, A. L. Extended virulence genotypes of *Escherichia coli* strains from patients with urosepsis in relation to phylogeny and host compromise. *J Infect Dis*. **2000**;181(1), 261–272. DOI: 10.1086/315217.
36. Russo, T. A.; Olson, R.; Fang, C. T.; Stoesser, N.; Miller, M.; MacDonald, U.; Hutson, A.; Barker, J. H.; La Hoz, R. M.; Johnson, J. R. Identification of Biomarkers for Differentiation of Hypervirulent *Klebsiella pneumoniae* from Classical *K. pneumoniae*. *J Clin Microbiol*. **2018**; 56(9), e00776-18. https://doi.org/10.1128/JCM.00776-18.
82. Bialek-Davenet, S.; Criscuolo, A.; Ailloud, F.; Passet, V.; Nicolas-Chanoine, M. H.; Decré, D.; Brisse, S. Development of a multiplex PCR assay for identification of *Klebsiella pneumoniae* hypervirulent clones of capsular serotype K2. *J Med Microbiol*. **2014**; 63(Pt 12), 1608–1614. https://doi.org/10.1099/jmm.0.081448-0.
86. Poirel, L.; Walsh, T. R.; Cuvillier, V.; Nordmann, P. Multiplex PCR for detection of acquired carbapenemase genes. Diagn Microbiol Infect Dis. 2011;70(1), 119–123. https://doi.org/10.1016/j.diagmicrobio.2010.12.002
87. Mora, A.; Viso, S.; López, C.; Alonso, M.P.; García-Garrote, F.; Dabhi, G.; Mamani, R.; Herrera, A.; Marzoa, J.; Blanco, M.; et al. Poultry as reservoir for extraintestinal pathogenic *Escherichia coli* O45:K1:H7-B2-ST95 in humans. *Vet Microbiol*. **2013**; 167, 506-512. https://doi.org/10.1016/j.vetmic.2013.08.007.
88. Saladin, M.; Cao, V.T.; Lambert, T.; Donay, J.L.; Herrmann, J.L.; Ould-Hocine, Z.; Verdet, C.; Delisle, F.; Philippon, A.; Arlet, G. Diversity of CTX-M beta-lactamases and their promoter regions from Enterobacteriaceae isolated in three Parisian hospitals. *FEMS Microbiol Lett*. **2002**;209, 161-168. https://doi.org/10.1111/j.1574-6968.2002.tb11126.x
89. Simarro, E.; Navarro, F.; Ruiz, J.; Miró, E.; Gómez, J.; Mirelis, B. *Salmonella enterica* serovar *virchow* with CTX-M-like beta-lactamase in Spain. *J Clin Microbiol*. **2000**; 38, 4676-4678. https://doi.org/10.1128/jcm.38.12.4676-4678.2000.
90. García-Meniño, I., García, V., Mora, A., Díaz-Jiménez, D., Flament-Simon, S.C., Alonso, M.P., Blanco, J.E., Blanco, M., Blanco, J. Swine enteric colibacillosis in Spain: pathogenic potential of *mcr-1* ST10 and ST131 *E. coli* isolates. *Front Microbiol*. **2018**; 9:2659. https://doi.org/10.3389/fmicb.2018.02659.
91. Pérez-Pérez, F. J.; Hanson, N. D. Detection of plasmid-mediated AmpC beta-lactamase genes in clinical isolates by using multiplex PCR. *J Clin Microbiol*. **2002**;40(6), 2153–2162. https://doi.org/10.1128/jcm.40.6.2153-2162.2002.
92. . Rebelo, A. R.; Bortolaia, V.; Kjeldgaard, J. S.; Pedersen, S. K.; Leekitcharoenphon, P.; Hansen, I. M.; Hendriksen, R. S. Multiplex PCR for detection of plasmid mediated colistin resistance determinants,

mcr-1, *mcr-2*, *mcr-3*, *mcr-4* and *mcr-5* for surveillance purposes. *Euro Surveill*. **2018**; 23(6):17-00672. https://doi.org/10.2807/1560-7917.ES.2018.23.6.17-00672.

93. Borowiak, M.; Fischer, J.; Hammerl, J. A.; Hendriksen, R. S.; Szabo, I.; Malorny, B. Identification of a novel transposon-associated phosphoethanolamine transferase gene, *mcr-5*, conferring colistin resistance in d-tartrate fermenting *Salmonella enterica* subsp. *enterica* serovar Paratyphi B. *J Antimicrob Chemother*. **2017**; 72(12), 3317–3324. https://doi.org/10.1093/jac/dkx327.
94. Le Bouguenec, C.; Archambaud, M.; Labigne, A. Rapid and specific detection of the pap, afa, and sfa adhesin-encoding operons in uropathogenic *Escherichia coli* strains by polymerase chain reaction. *J Clin Microbiol*. **1992**; 30(5), 1189–1193. https://doi.org/10.1128/jcm.30.5.1189-1193.1992.
95. Lescat, M.; Clermont, O.; Woerther, P.L.; Glodt, J.; Dion, S.; Skurnik, D.; Djossou, F.; Dupont, C.; Perroz, G.; Picard, B.; et al. Commensal *Escherichia coli* strains in Guiana reveal a high genetic diversity with host-dependant population structure. *Environ Microbiol Rep*. **2013**; 5, 49-57. https://doi.org/10.1111/j.1758-2229.2012.00374.x.
96. Mora, A.; Herrrera, A.; Lopez, C.; Dahbi, G.; Mamani, R.; Pita, J.M.; Alonso, M.P.; Llovo, J.; Bernardez, M.I.; Blanco, J.E.; et al. Characteristics of the Shiga-toxin-producing enteroaggregative *Escherichia coli* O104:H4 German outbreak strain and of STEC strains isolated in Spain. *Int Microbiol*. **2011**; 4, 121-141. https://doi.org/10.2436/20.1501.01.142.
97. Johnson, J. R.; Russo, T. A.; Tarr, P. I.; Carlino, U.; Bilge, S. S.; Vary, J. C.; Stell, A. L. Molecular epidemiological and phylogenetic associations of two novel putative virulence genes, *iha* and *iroN* (*E. coli*), among *Escherichia coli* isolates from patients with urosepsis. *Infect Immun*. **2000**; 68(5), 3040–3047. https://doi.org/10.1128/IAI.68.5.3040-3047.2000.
98. Johnson, J. R.; Gajewski, A.; Lesse, A. J.; Russo, T. A. Extraintestinal pathogenic *Escherichia coli* as a cause of invasive non urinary infections. *J Clin Microbiol*. **2003**;41(12), 5798–5802. https://doi.org/10.1128/JCM.41.12.5798-5802.2003.
99. Yamamoto, S.; Terai, A.; Yuri, K.; Kurazono, H.; Takeda, Y.; Yoshida, O. Detection of urovirulence factors in *Escherichia coli* by multiplex polymerase chain reaction. *FEMS Immunol Med Microbiol*. **1995**; 12(2), 85–90. https://doi.org/10.1111/j.1574-695X.1995.tb00179.x.
100. Tóth, I.; Hérault, F.; Beutin, L.; Oswald, E. Production of cytolethal distending toxins by pathogenic *Escherichia coli* strains isolated from human and animal sources: establishment of the existence of a new cdt variant (Type IV). *J Clin Microbiol*. **2003**; 41(9), 4285–4291. https://doi.org/10.1128/JCM.41.9.4285-4291.2003.
101. Moulin-Schouleur, M.; Schouler, C.; Tailliez, P.; Kao, M.-R.; Brée, A.; Germon, P.; Oswald, E.; Mainil, J.; Blanco, M.; Blanco, J. Common virulence factors and genetic relationships between O18:K1:H7 *Escherichia coli* isolates of human and avian origin. *J Clin Microbiol*. **2006**; 44(10), 3484–3492. https://doi.org/10.1128/JCM.00548-06.
102. Johnson, J. R.; O'Bryan, T. T. Detection of the *Escherichia coli* group 2 polysaccharide capsule synthesis Gene *kpsM* by a rapid and specific PCR-based assay. *J Clin Microbiol*. **2004**;42(4), 1773–1776. https://doi.org/10.1128/JCM.42.4.1773-1776.2004.
112. Wirth, T., Falush, D., Lan, R., Colles, F., Mensa, P., Wieler, L. H., Achtman, M. Sex and virulence in *Escherichia coli*: an evolutionary perspective. *Mol Microbiol*. **2006**; 60(5), 1136–1151. DOI: 10.1111/j.1365-2958.2006.05172.x.
